# High Residual Platelet Reactivity during Aspirin Therapy in Patients with Non-St Segment Elevation Acute Coronary Syndrome: Comparison Between Initial and Late Phases

**DOI:** 10.5935/abc.20190146

**Published:** 2019-09

**Authors:** Marianna Deway Andrade Dracoulakis, Paul Gurbel, Marco Cattaneo, Herlon Saraiva Martins, José Carlos Nicolau, Roberto Kalil Filho

**Affiliations:** 1 Hospital da Bahia - Instituto de Ensino e Pesquisa, Salvador, BA - Brazil; 2 Sinai Hospital of Baltimore - Sinai Center for Thrombosis Research, Baltimore - EUA; 3 Universita Degli Studi Di Milano - Unita di Medicina III, Milão - Itália; 4 Universidade de São Paulo - Faculdade de Medicina Hospital das Clínicas, São Paulo, SP - Brazil

**Keywords:** Acute Coronary Syndrome, Platelet Aggregation/drug effects, Myocardial Ischemia, Aged, aspirin/therapeutic use, Aspirin/adverse effects

## Abstract

**Background:**

High platelet reactivity (HPR) during therapy with acetylsalicylic acid (ASA) is a poor prognostic factor in acute coronary syndromes (ACS). The prevalence of HPR during ACS is greater than that reported in stable diseases. However, it is unclear whether this prevalence of HPR is a transient phenomenon or a characteristic of this high-risk population.

**Objective:**

The main objective is to compare the effects of ASA on platelet function in the initial and late phases of ACS in a single population. Secondary objectives are: correlation between the tests between themselves and the relationship between the tests and the variation of the inflammatory markers (C-reactive protein and interleukin-6).

**Methods:**

Seventy patients with non-ST segment elevation (NSTE) ACS in use of 100-200 mg of ASA per day for at least 7 days were prospectively studied. Platelet function was assessed in the first 48 hours and subsequently after 3 months using four methods: VerifyNow™ (VFN), whole blood platelet aggregation (WBPA) with arachidonic acid (AA) and collagen as agonists, and platelet function analyzer (PFA). The level of statistical significance considered was < 0.05.

**Results:**

According to the more specific methods (WBPA with AA and VFN), the incidence of HPR was significantly higher in the early phase than in the late phase: WBPA with AA: 31% versus 13%, p = 0.015; VFN: 32% versus 16%, p = 0.049. The other methods tested, which were less specific for ASA, did not show significant differences between phases. The correlation between the methods was weak or moderate (r ranging from 0.3 to 0.5, p < 0.05), and there were no significant associations between HPR and inflammatory markers.

**Conclusion:**

The prevalence of HPR during AAS therapy, assessed by specific methods for cyclooxygenase 1 (COX-1), is higher during the acute phase than in the late phase of NSTE ACS.

## Introduction

Acetylsalicylic acid (ASA) is widely used as first-line antiplatelet therapy for acute coronary syndromes (ACS) and is recommended by the guidelines of the American Heart Association and the American College of Cardiology,^1^ European Society of Cardiology^2^ and the Brazilian Society of Cardiology^3^ for patients with non-ST segment elevation acute coronary syndromes (NSTE ACS).

AAS has been tested with proven efficacy in several randomized clinical trials across the spectrum of both acute and chronic coronary artery disease.^4-7^ However, some studies have demonstrated high variability in the individual antiplatelet response to ASA in different populations and scenarios.^8^ This variability may contribute, at least in part, to the high rate of recurrence of ischemic events in patients with coronary artery disease.^9,10^

The prevalence of high platelet reactivity (HPR) in patients using ASA depends, among other factors, on the laboratory test and cut-off point used, as well as on the clinical picture. In patients with chronic arterial disease, the prevalence ranges from 0 to 57% (24%, on average).^10-13^ More importantly, patients with HPR have been described to have a poorer clinical outcome, with a higher incidence of serious cardiovascular events, including mortality.^10,11,14^

In ACS, the estimated prevalence of HPR is supposedly higher.^15^ Previous studies have suggested that atherosclerotic load and systemic inflammation may have a significant influence on platelet reactivity.^16,17^ However, it is not clear whether this high prevalence of HPR is a transient acute phase phenomenon or a permanent characteristic of this high risk population, since, to the best of our knowledge, no study has analyzed the response to ASA during the acute and chronic phases in the same population. The present study was designed to give a definitive answer on this important question.

## Methods

### Study population

Prospective inclusion of 70 consecutive patients admitted to the emergency department (ED) of a tertiary cardiology hospital with a diagnosis of NSTE ACS, with initial evaluation at admission (acute phase) and subsequently 3 months after discharge (late phase). Patients were considered eligible for inclusion if aged ≥ 18 years, had been diagnosed with unstable angina or non-ST segment elevation myocardial infarction within the first 48 hours of clinical onset, and were using 100 mg to 200 mg of AAS for at least 7 days prior to the event.

The main exclusion criteria were the use of another antiplatelet agent in addition to ASA, oral or parenteral anticoagulation, percutaneous coronary intervention (PCI) in the last 30 days or myocardial revascularization surgery in the last 90 days. Other exclusion criteria were hemoglobin < 10 g/dL; platelets < 100,000/mm^3^ or > 500,000/mm^3^; creatinine clearance < 30 mL/min; decompensated heart failure (Killip III or IV); current use of inotropes or vasopressors; and known hematological or neoplastic diseases.

### Model

Patients were evaluated at two different moments: initially, at admission to ED, prior to the administration of any other antithrombotic treatment except ASA, and 3 months after hospital discharge, when they should also be on ASA as the only antiplatelet agent. At each evaluation, patients were evaluated and interviewed, and underwent blood collection 1 to 4 hours after the use of ASA. Adherence to ASA treatment was systematically evaluated during face-to-face medical interviews. The study is in accordance with the Helsinki Declaration and was approved by the local Ethics Committee; patients provided their informed consent.

### Objectives

The primary objective of the study was to compare platelet aggregation in patients with NSTE ACS in the acute phase (the first 48 hours of the clinical picture) in relation to the late phase (3 months after) using four different methods of evaluating platelet aggregation: VerifyNow™ aspirin (VFN) (Accumetrics, Inc., San Diego, California, USA); whole blood platelet aggregation (WBPA) using arachidonic acid (AA) (Sigma-Aldrich, Saint Louis, Missouri, USA) and collagen (Chrono-Log®; Chrono-Log Co., Havertown, Pennsylvania, USA); PFA-100® Platelet Function Analyzer with collagen/ADP cartridge (COL/EPI) (Siemens Healthcare Diagnostics, Newark, Delaware, USA). Secondary objectives were the correlation between the four tests in the acute phase and the relationship between each of the tests with inflammatory markers (C-reactive protein and interleukin-6).

### Blood collection

All blood samples were collected through antecubital venous puncture with a 21 gauge needle between 10:00 am and 1:00 pm. The four tests were performed within two hours of the collection.

### Definition of HPR

The cut-off values used to define HPR were: PFA-100^®^, closure time (CT) < 150 seconds;^18^ VFN, aspirin reaction units (ARU) ≥ 550 (according to the manufacturer); WBPA with AA, Ω ≥ 3;^19^ WBPA with collagen, Ω ≥ 10.^20^

### Statistical analysis

The sample size was calculated based on the expected mean result of the PFA-100^®^ test, which was 191 seconds ± 100^21^ during the acute phase, and the 25-second reduction estimate of that value in the chronic phase. According to the McNemar test, with 80% power and alpha of 0.05, 70 patients were required. The continuous variables were evaluated for their distribution (Gaussian or not) using the Kolmogorov-Smirnov test.

Parametric continuous variables were presented as mean ± standard deviation, and nonparametric variables as medians and interquartile ranges (25-75). The unpaired Mann-Whitney (non-Gaussian variables) or Student’s T (Gaussian variables) tests were used with the Welch correction when indicated. When comparing two different moments, the Wilcoxon test was used for the non-Gaussian variables and the paired Student’s T for Gaussian samples. The categorical variables were presented in relative and absolute frequencies. Contingency distribution tables were analyzed using the chi-square test and Fisher’s exact test. Analysis of the correlation between the tests was done with Spearman's correlation coefficient. Values of p < 0.05 were considered statistically significant. The software used was SPSS (IBM Corporation), version 11.

## Results

### Patients’ characteristics

The demographic and baseline characteristics of the patients are summarized in Table 1. Almost half of the patients reported a previous history of diabetes. The majority (64%) had a classification for thrombolysis in myocardial infarction (TIMI) with risk for non-ST segment elevation ACS equal to 3 or 4 on admission. All patients were on 100 to 200 mg ASA as the only antiplatelet agent in the last 7 days prior to the collection of the tests, both in the acute phase and in the late phase.

**Table 1:** Demographic and baseline characteristics of patients

Number of patients	70
Age, years (mean ± SD)	64.2 ± 9.7
Female, n (%)	38 (54.3)
**Medical history**	
Diabetes mellitus, n (%)	34 (48.6)
Hypertension, n (%)	61 (87.1)
Dyslipidemia, n (%)	58 (82.9)
Current smoking, n (%)	11 (15.7)
Obesity, n (%)	16 (22.9)
Family history of CAD, n (%)	28 (40)
AMI, n (%)	41 (58.6)
SMR or PCI, n (%)	38 (54.3)
CHF, n (%)	6 (8.6)
**Type of ACS**	
Unstable angina, n (%)	54 (77.1)
NSTE AMI, n (%)	16 (22.9)
**TIMI risk score**	
0 to 2, n (%)	15 (21)
3 to 4, n (%)	45 (64)
≥ 5 (%)	10 (15)
**Previously used medications**	
PPIs, n (%)	32 (45.7)
Beta-blockers, n (%)	55 (78.6)
Calcium channel blockers, n (%)	10 (15)
ACEIs/ARBs, n (%)	45 (64.3)
Aldosterone antagonists, n (%)	3 (4.3)
Laboratory tests	Median (25^th^/75^th^)
Hemoglobin, g/dL	13.7 (12.8/14.7)
Leukocytes × 1.000/mm^3^	8.0 (6.5/9.2)
Platelets × 1.000/mm^3^	220 (179/273)
Creatinine, g/dL	1.0 (0.9/1.2)

ARBs: angiotensin receptor blockers; SMR: Surgical myocardial revascularization; CAD: coronary artery disease; AMI: acute myocardial infarction; PPIs: proton pump inhibitors; CHF: congestive heart failure; PCI: percutaneous coronary intervention; ACEIs: angiotensin converting enzyme inhibitors; ACS: acute coronary syndrome; NSTE: non-ST segment elevation; TIMI: thrombolysis in myocardial infarction.

### Primary objective

**Figure 1:**
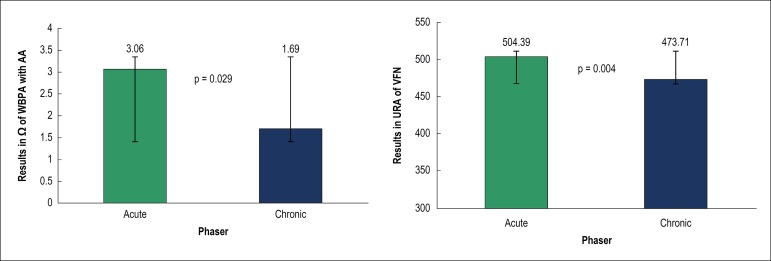
Comparison of COX-1-specific tests (WBPA with AA and VFN) between the acute and late phases. WBPA: whole blood platelet aggregation; AA: arachidonic acid; VFN: VerifyNow™; URA: units of reaction to acetylsalicylic acid.

Platelet aggregation tests were divided into COX-1-specific (WBPA with AA and VFN) and COX-1-nonspecific (WBPA with collagen and PFA-100®). COX-1-specific tests were associated with higher platelet reactivity in the acute phase, compared to the late phase (Figure 1). Comparisons between the phases by the nonspecific COX-1 tests did not show significant differences (PFA = 215.9 ± 83.75 seconds *versus* 200.51 ± 84.63 seconds, respectively, in the acute and late phases, p = 0.233; WBPA with collagen, 7.19 ± 5.64 Ω *versus* 6.46 ± 5.09 Ω, p = 0.658).

When the results were categorized according to pre-established cutoff values for HPR diagnosis (Table 2), COX-1-specific tests were associated with significant differences between the acute and late phases (WBPA with AA, 31.4% *versus* 12.8%, p = 0.015; VFN, 32.1% *versus* 16%, p = 0.049), whereas nonspecific tests did not show significant differences (PFA, 34.2% *versus* 40%, p = 0.50; WBPA with collagen, 33.8% *versus* 30.8%, p = 0.86).

**Table 2:** Comparison of HPR by different platelet tests between the acute and late phases

Test	Acute Phase	Late Phase	p
	HPR	HPR	
PFA	34.2%	40%	0.503
WBPA with AA	31.4%	12.8%	0.015
VFN	32.1%	16%	0.049
WBPA with Col	33.8%	30.8%	0.860

WBPA: whole blood platelet aggregation; AA: arachidonic acid; Col: collagen; PFA: Platelet Function Analyzer (PFA-100^®^); VFN: VerifyNow™; p: p value.

### Secondary objectives

#### Correlation between platelet tests

In the acute phase, the analyzed methods correlated significantly (Table 3). However, the magnitude of this correlation was only moderate (r > 0.4) between WBPA with AA and WBPA with collagen. The correlation between the other methods was only weak (r > 0.2 and < 0.4).

**Table 3:** Correlation between platelet tests in the acute phase

		WBPA with AA	WBPA with Col	VFN
PFA	r_s_	–0.429*	–0.281*	–0.279*
WBPA with AA	r_s_		0.498*	0.393*
WBPA with Col	r_s_			0.318*

* p < 0.05, AA: arachidonic acid; Col: collagen; WBPA: whole blood platelet aggregation; PFA: PFA-100®; r_s_: Spearman correlation coefficient; VFN: VerifyNow™.

#### Variation of inflammatory markers and platelet reactivity between acute and late phases

**Figure 2:**
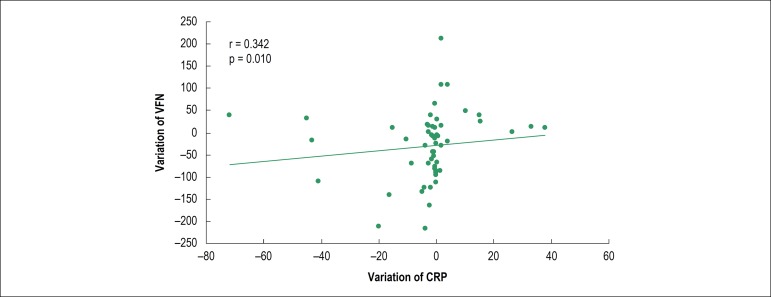
Correlation between the variation of CRP and VFN (acute/late). CRP: C-reactive protein; VFN: VerifyNow™; r: Spearman’s coefficient.

C-reactive protein (CRP) levels differed significantly between the acute and late phases [median CRP = 2.84 mg/dL (1.54 to 8.41) *versus* 1.41 mg/dL (0.73 to 5.64), p = 0.006], whereas interleukin-6 (IL-6) did not differ between the two phases [median IL-6 = 2.1 pg/mL (2.0 a 5.68) *versus* 2.0 pg/mL (2.0 to 3.25), p = 0.110]. When CRP (acute/late) variation was compared to the variation of the methods in the two phases analyzed, a weak but significant correlation (Figure 2) was demonstrated between CRP and VFN (r = 0.29, p= 0.03).

## Discussion

Our data demonstrate significant differences in response to ASA during the acute and late phases of acute coronary disease.

Previous studies have unequivocally documented that ASA reduces the occurrence of cardiovascular events in patients with CAD.^4-7^ Even with the advent of the new antiplatelet agents that act by blocking the P2Y12 receptor, the role of ASA remains unchanged as it is considered, in all guidelines, a routine treatment in this population.^1-2^ However, it has been well established that there is significant variability in residual platelet function during ASA therapy, especially in the context of ACS, in which the prevalence of HPR is more evident.^8,17^ The reason for this variability is not fully understood. One hypothesis is that HPR is present in a subpopulation of patients with chronic CAD, leading to a decrease in the efficacy of ASA and, as a consequence, increasing the likelihood of developing ischemic cardiovascular events. Another hypothesis is that HPR develops during the acute ischemic episode, as a consequence of the increase in platelet reactivity due to phenomena occurring in the acute phase (increased inflammatory activity, increased rate of platelet renewal, activation of the coagulation system, among others).

To our knowledge, this study was the first to test both hypotheses in the same population of patients with NSTE ACS. Our results showed that, for most patients, HPR is labile, with a higher prevalence observed during the acute phase compared to the late phase. These results are consistent with the data reported by Hobikoglu et al.,^21^ who analyzed two different populations (one group of patients hospitalized with ACS and another group of patients with chronic CAD).

The present demonstrations can have a significant therapeutic impact, since approximately one third of our patients showed HPR during the initial phase of ACS, and new regimens, including change of dosage and use of more potent antiplatelet agents, may be proposed to reduce the risk of ischemic events. Neubauer et al.^22^ evaluated a therapeutic regimen of dose escalation of ASA and clopidogrel in patients with ACS or unstable angina undergoing PCI and considered nonresponders by WBPA with AA and adenosine diphosphate (ADP). Patients considered nonresponders to ASA were treated with increasing doses of 100 mg to 300 mg per day, and up to 500 mg, if necessary, with improved therapeutic response.

On the other hand, our data demonstrate that, although there is a significant decrease in the incidence of HPR during the chronic phase, a significant percentage of the population still present HPR at this stage.

The high rate of platelet turnover that occurs in several situations (including ACS) could be one of the explanations for our findings; however, this mechanism was not analyzed in the present study. As demonstrated in previous studies in diabetic patients in the postoperative period of cardiac surgery,^23-25^ the number of circulating immature platelets increases as a consequence of increased platelet consumption, leading to an exponential increase in the platelet turnover rate. In a study by Dillinger et al.,^26^ comparing different doses of ASA twice daily in diabetic patients with CAD and at least one risk factor, twice daily use of the drug reduced HPR rate when compared to the same dose administered once a day. However, in the CURRENT study, the use of a double dose of ASA showed no benefit when compared to the conventional dose.^27^

Another possibility would be the influence of the inflammatory process, which is characteristic of the acute phase, on platelet function, resulting in increased platelet activation and increased HPR in response to ASA. In the present study, there was a significant but weak association between inflammation and platelet reactivity, analyzed by PCR and VNF, respectively (r = 0.293, p = 0.03). In a stable CAD population, Bernlochner et al.^28^ showed a significant, positive and independent association between CRP levels and platelet aggregation, which were assessed by WBPA with ADP. Similarly, Tantry et al.^29^ reported a significant correlation between inflammatory markers (including CRP), markers of hypercoagulability and platelet function in different CAD spectra (asymptomatic, stable CAD and ACS). However, contrary to these findings, Ziegler et al.^30^ demonstrated that in patients with peripheral arterial disease, there was no significant correlation between CRP and platelet aggregation measured by PFA-100^®^. These conflicting results can be attributed, at least in part, to methodological differences.

In our study, different methods of determining platelet function were used simultaneously. The correlation between the tests during the acute phase was significant, but the magnitude of these correlations was only weak or moderate. Unexpectedly, even the methods classified as COX-1-specific showed medium to low correlation with each other. These findings are consistent with findings from previous studies: Lordkipanidzé et al.^19^ studied 201 patients with stable CAD undergoing aspirin therapy^19^ using six different tests. The prevalence of HPR varied from 4% when analyzed by optical aggregometry with AA, to 59.5%, when analyzed by PFA-100^®^ (COL/EPI). In this study, as in ours, there were weak correlations between methods for determining platelet function, including COX-1 specific methods. The present study was the first to analyze different methods of platelet aggregation during the acute and late phases, in the same population of patients with NSTE ACS.

In summary, our findings may have important therapeutic implications in demonstrating that one-third of the patients showed HPR in the acute phase, leading to the hypothesis that new dosing regimens should be tested in this population. In addition, despite the fact that there is a significant decrease in the incidence of HPR during the chronic phase, a significant percentage of the population still presents HPR at this stage.

### Study limitations

Firstly, our study had a relatively small sample size, but it was adequate to assess the primary outcome. However, the secondary results should be considered as hypothesis generators and interpreted with caution. Secondly, all patients were on chronic ASA use at a dose of 100 mg/day to 200 mg/day, but individual doses were not collected and may have influenced the results obtained.^25^ Lastly, in recent times, the role of young (immature) platelets has been valued; if they had been assessed in the present study (which was not done), they could have added important information.

## Conclusion

In conclusion, the prevalence of HPR during ASA therapy measured by COX-1-specific methods is higher during the acute phase than in the late phase of patients with non-ST segment elevation ACS. However, the relationship between inflammation as indicated by CRP and IL-6 and platelet reactivity in these two phases is weak, suggesting that the variability in the inflammation state may not play a role in the temporal changes in platelet reactivity in this population.

## References

[1] Amsterdam EA, Wenger NK, Brindis RG, Casey DE Jr, Ganiats TG, Holmes DR Jr (2014). 2014 AHA/ACC guideline for the management of patients with
non-ST-elevation acute coronary syndromes: a report of the American College
of Cardiology/American Heart Association Task Force on Practice
Guidelines. Circulation.

[2] Roffi M, Patrono C, Collet JP, Mueller C, Valgimigli M, Andreotti F (2016). 2015 ESC Guidelines for the management of acute coronary
syndromes in patients presenting without persistent ST-segment elevation:
Task Force for the Management of Acute Coronary Syndromes in Patients
Presenting without Persistent ST-Segment Elevation of the European Society
of Cardiology (ESC). Eur Heart J.

[3] Nicolau JC, Timerman A, Marin-Neto JA, Piegas LS, Barbosa CJ, Franci A (2014). Guidelines of Sociedade Brasileira de Cardiologia for Unstable
Angina and Non-ST-Segment Elevation Myocardial Infarction (II Edition, 2007)
2013-2014 Update. Arq Bras Cardiol.

[4] Lewis HD Jr, Davis JW, Archibald DG, Steinke WE, Smitherman TC, Doherty JE (1983). Protective effects of aspirin against acute myocardial infarction
and death in men with unstable angina: Results of a Veterans Administration
Cooperative Study. N Engl J Med.

[5] Théroux P, Ouimet H, McCans J, Latour JG, Joly P, Lévy G (1988). Aspirin, heparin, or both to treat acute unstable
angina. N Engl J Med.

[6] ISIS-2 (Second International Study of Infarct Survival)
Collaborative Group (1988). Randomised trial of intravenous streptokinase, oral aspirin,
both, or neither among 17,187 cases of suspected acute myocardial
infarction: ISIS-2. Lancet.

[7] Antithrombotic Trialists' Collaboration (2002). Collaborative meta-analysis of randomised trials of antiplatelet
therapy for prevention of death, myocardial infarction, and stroke in high
risk patients. BMJ.

[8] Hovens MM, Snoep JD, Eikenboom JC, van der Bom JG, Mertens BJ, Huisman MV (2007). Prevalence of persistent platelet reactivity despite use of
aspirin: a systematic review. Am Heart J.

[9] Baigent C, Blackwell L, Collins R, Emberson J, Godwin J, Antithrombotic Trialists' (ATT) Collaboration (2009). Aspirin in the primary and secondary prevention of vascular
disease: collaborative meta-analysis of individual participant data from
randomised trials. Lancet.

[10] Chen WH, Cheng X, Lee PY, Ng W, Kwok JY, Tse HF (2007). Aspirin resistance and adverse clinical events in patients with
coronary artery disease. Am J Med.

[11] Eikelboom JW, Hirsh J, Weitz JI, Johnston M, Yi Q, Yusuf S (2002). Aspirin-resistant thromboxane biosynthesis and the risk of
myocardial infarction, stroke, or cardiovascular death in patients at high
risk for cardiovascular events. Circulation.

[12] Le Quellec S, Bordet JC, Negrier C, Dargaud Y (2016). Comparison of current platelet functional tests for the
assessment of aspirin and clopidogrel response. Thromb Haemost.

[13] Gurbel PA, Bliden KP, DiChiara J, Newcomer J, Weng W, Neerchal NK (2007). Evaluation of dose-related effects of aspirin on platelet
function: results from the Aspirin-Induced Platelet Effect (ASPECT)
study. Circulation.

[14] Gori AM, Grifoni E, Valenti R, Giusti B, Paniccia R, Parodi G (2016). High on-aspirin platelet reactivity predicts cardiac death in
acute coronary syndrome patients undergoing PCI. Eur J Intern Med.

[15] Hobikoglu GF, Norgaz T, Aksu H, Ozer O, Erturk M, Nurkalem Z (2005). High frequency of aspirin resistance in patients with acute
coronary syndrome. Tohoku J Exp Med.

[16] Aksu K, Donmez A, Keser G (2012). Inflammation-induced thrombosis: mechanisms, disease associations
and management. Curr Pharm Des.

[17] Muhlestein JB (2010). Effect of antiplatelet therapy on inflammatory markers in
atherothrombotic patients. Thromb Haemost.

[18] Buyukasik Y, Karakus S, Goker H, Haznedaroglu IC, Ozatli D, Sayinalp N (2002). Rational use of the PFA-100 device for screening of platelet
function disorders and von Willebrand disease. Blood Coagul Fibrinolysis.

[19] Lordkipanidzé M, Pharand C, Schampaert E, Turgeon J, Palisaitis DA, Diodati JG (2007). A comparison of six major platelet function tests to determine
the prevalence of aspirin resistance in patients with stable coronary artery
disease. Eur Heart J.

[20] Ivandic BT, Giannitsis E, Schlick P, Staritz P, Katus HA, Hohlfeld T (2007). Determination of aspirin responsiveness by use of whole blood
platelet aggregometry. Clin Chem.

[21] Hobikoglu GF, Norgaz T, Aksu H, Ozer O, Erturk M, Destegul E (2007). The effect of acetylsalicylic acid resistance on prognosis of
patients who have developed acute coronary syndrome during acetylsalicylic
acid therapy. Can J Cardiol.

[22] Neubauer H, Kaiser AF, Endres HG, Krüger JC, Engelhardt A, Lask S (2011). Tailored antiplatelet therapy can overcome clopidogrel and
aspirin resistance - the Bochum clopidogrel and aspirin plan (BOCLA-Plan) to
improve antiplatelet therapy. BMC Med.

[23] Tschoepe D, Roesen P, Esser J, Schwippert B, Nieuwenhuis HK, Kehrel B (1991). Large platelets circulate in an activated state in diabetes
mellitus. Semin Thromb Hemost.

[24] Golanski J, Chlopicki S, Golanski R, Gresner P, Iwaszkiewicz A, Watala C (2005). Resistance to aspirin in patients after coronary artery bypass
grafting is transient: impact on the monitoring of aspirin antiplatelet
therapy. Ther Drug Monit.

[25] Rocca B, Santilli F, Pitocco D, Mucci L, Petrucci G, Vitacolonna E (2012). The recovery of platelet cyclooxygenase activity explains
interindividual variability in responsiveness to low-dose aspirin in
patients with and without diabetes. J Thromb Haemost.

[26] Dillinger JG, Drissa A, Sideris G, Bal dit Sollier C, Voicu S, Silberman SM (2012). Biological efficacy of twice daily aspirin in type 2 diabetic
patients with coronary artery disease. Am Heart J.

[27] Mehta SR, Tanguay JF, Eikelboom JW, Jolly SS, Joyner CD, Granger CB (2010). Double-dose versus standard-dose clopidogrel and high-dose versus
low-dose aspirin in individuals undergoing percutaneous coronary
intervention for acute coronary syndromes (CURRENT-OASIS 7): a randomised
factorial trial. Lancet.

[28] Bernlochner I, Steinhubl S, Braun S, Morath T, Jaitner J, Stegherr J (2010). Association between inflammatory biomarkers and platelet
aggregation in patients under chronic clopidogrel treatment. Thromb Haemost.

[29] Tantry US, Bliden KP, Suarez TA, Kreutz RP, Dichiara J, Gurbel PA (2010). Hypercoagulability, platelet function, inflammation and coronary
artery disease acuity: Results of the Thrombotic Risk Progression (TRIP)
Study. Platelets.

[30] Ziegler S, Alt E, Brunner M, Speiser W, Minar E (2005). Influence of systemic inflammation on the interpretation of
response to antiplatelet therapy, monitored by PFA-100. Semin Thromb Hemost.

